# Morphological Changes in the Myocardium of Patients with Post-Acute Coronavirus Syndrome: A Study of Endomyocardial Biopsies

**DOI:** 10.3390/diagnostics13132212

**Published:** 2023-06-29

**Authors:** Igor Makarov, Sofya Mayrina, Taiana Makarova, Tatiana Karonova, Anna Starshinova, Dmitry Kudlay, Lubov Mitrofanova

**Affiliations:** 1 Almazov National Medical Research Centre, 197341 St. Petersburg, Russiamakarova_ta@almazovcentre.ru (T.M.);; 2 Department of Pharmacology, Institute of Pharmacy, I.M. Sechenov First Moscow State Medical University, 119435 Moscow, Russia; 3 Institute of Immunology, 115478 Moscow, Russia

**Keywords:** PASC, post-acute sequelae of SARS-CoV-2 infection, COVID-19-associated myocarditis, inflammatory cardiomyopathy, angiopathy, CD68+ macrophages

## Abstract

The clinical manifestation study of post-acute sequelae of SARS-CoV-2 infection (PASC) has shown a lack of knowledge regarding its morphology and pathogenesis. The aim of this research was to investigate morphological manifestations of PASC in the myocardium. Materials and Methods: The study included 38 patients requiring endomyocardial biopsy (EMB) during the post-acute phase of coronavirus infection and a control group including patients requiring EMB prior to the SARS-CoV-2 pandemic. The patients’ clinical and laboratory data were analyzed. Histological examination and immunohistochemistry (IHC) of the myocardial tissue was conducted with antibodies to CD3, CD68, HLA-DR, MHC1, C1q, VP1 enteroviruses, spike protein SARS-CoV-2, Ang1, von Willebrand factor (VWF), and VEGF. The morphometric analysis included counting the mean number of inflammatory infiltrate cells per mm^2^ and evaluating the expression of SARS-CoV-2 spike protein, HLA-DR, MHC1, C1q, Ang1, VWF, and VEGF using a scoring system. If the expression of SARS-CoV-2 spike protein was >3 points, an additional IHC test with antibodies to ACE2, CD16 as well as RT-PCR testing of the myocardial tissue were performed. For two patients, immunofluorescence tests of the myocardial tissue were performed using antibody cocktails to SARS-CoV-2 spike protein/CD16, SARS-CoV-2 spike protein/CD68, CD80/CD163. The statistical data analysis was carried out using the Python programming language and libraries such as NumPy, SciPy, Pandas, and Matplotlib. Results: The study demonstrated a significant increase in the number of CD68+ macrophages in the myocardium of PASC patients compared to patients who did not have a history of COVID-19 (*p* = 0.014 and *p* = 0.007 for patients with and without myocarditis, respectively), predominantly due to M2 macrophages. An increase in the number of CD68+ macrophages was more frequently observed in patients with shorter intervals between the most recent positive SARS-CoV-2 PCR test and the time of performing the EMB (r = −0.33 and r = −0.61 for patients with and without myocarditis, respectively). The expression scores of Ang1, VEGF, VWF, and C1q in PASC patients did not significantly differ from those in EMB samples taken before 2019. Conclusion: The myocardium of PASC patients demonstrated a significant increase in the number of CD68+ macrophages and a decrease in the expression of markers associated with angiopathy. No evidence of coronavirus-associated myocarditis was observed in any PASC patient.

## 1. Introduction

The incidence of COVID-19 is currently decreasing, and the focus is shifting to the health issues of COVID-19 survivors. Although most people fully recover clinically and their lives return to normal within the first 1–4 weeks from the onset of the disease, some patients experience long-term manifestations that require additional attention and investigations [1]. The persistence of symptoms or the development of new signs of illness 4 weeks after the onset of COVID-19 is commonly referred to as Post-Acute Sequelae of SARS-CoV-2 infection (PASC) [2].

The prevalence of PASC is estimated to be 10% among COVID-19 survivors, according to some studies [2]. The diagnostic criteria for PASC include the presence of one or more persistent clinical symptoms from the PASC symptom complex that could not be explained by the patient’s comorbidities. The symptom complex includes persistent fatigue, muscle and joint pains, decreased exercise tolerance, persistent low-grade fever, shortness of breath, palpitations, irregular heartbeat, sudden fainting, cough, chest pain, sleep disturbances, depression, diffuse alopecia, prolonged loss of taste and smell, skin rashes of various types, morphological changes affecting peripheral blood cells, impaired immune response, and sleep disorders [3,4,5,6].

The aim of this study was to investigate the cardiac manifestations of PASC. Despite a significant amount of published data on the characteristics and prevalence of this syndrome, our understanding of its pathophysiology and outcomes for patients is still incomplete. In particular, there is a lack of morphological data confirming structural changes that are typical for this syndrome. In our study, we aimed at filling this gap by providing new knowledge and data to improve the diagnosis and treatment of patients with PASC.

## 2. Materials and Methods

### 2.1. Patients

This cross-sectional retrospective study included 38 patients who were followed up at the Almazov National Medical Research Centre, and the inclusion criteria were the development/worsening of cardiovascular symptoms requiring the conduct of an endomyocardial biopsy (EMB) in patients with PASC. Symptoms requiring an EMB included: chest pain of unclear etiology, symptoms of myocarditis, progressive heart failure, ventricular rhythm disorders, syncope without any obvious causes. Exclusion criteria were as follows: cardiac sarcoidosis, purulent or granulomatous myocarditis found during the EMB, a small volume of biopsy specimens that did not allow for additional morphological studies. Patients were divided into two subgroups: patients without and with lymphocytic myocarditis. There were 29 patients in the subgroup with lymphocytic myocarditis and 9 patients in the subgroup without myocarditis. For each of these subgroups, comparison subgroups were formed: EMBs performed before 2019, including 8 and 33 patients without and with lymphocytic myocarditis, respectively (Table 1).

The severity of COVID-19 in all included patients was assessed based on the findings of chest CT scans performed during the acute phase of the infection. Other evaluated parameters included the time from COVID-19 to the EMB, the relationship between cardiovascular symptoms and COVID-19, the development or progression of chronic heart failure (HF) following COVID-19, heart rhythm disturbances and conduction abnormalities, left ventricular ejection fraction (LVEF) on admission, TAPSE, as well as the presence of comorbidities that could explain the onset or increased severity clinical symptoms.

### 2.2. Histological Examination, Immunohistochemistry, and SARS-CoV-2 RT-ddPCR Test

As part of the EMB procedure, conventional histological examination of the myocardial tissue with hematoxylin and eosin staining and immunohistochemistry (IHC) of the myocardial tissue with antibodies to CD3, CD68, HLA-DR, MHC1, C1q, VP1 enteroviruses were conducted. IHC with antibodies to the SARS-CoV-2 spike protein was performed in 27 cases (2 and 25 patients with and without myocarditis, respectively); in 22 cases (2 patients without myocarditis and 20 with myocarditis), IHC with antibodies to Ang1, von Willebrand factor (VWF), VEGF was performed, and in 6 cases (2 patients without myocarditis and 4 with myocarditis), IHC with antibodies to ACE2, CD16 was performed. Myocardial inflammation was diagnosed if there were ≥ 14 white blood cells/mm^2^ (including up to 4 monocytes/mm^2^) with the presence of CD3-positive T cells ≥ 7 cells/mm^2^ according to the position statement of the European Society of Cardiology Working Group on Myocardial and Pericardial Diseases [7].

Morphometric analysis included counting of the average number of inflammatory infiltrate cells per mm^2^ and evaluation of the expression of the SARS-CoV-2 spike protein, HLA-DR, MHC1, C1q, Ang1, VWF, and VEGF using a scoring system (1 point—expression on single cells and vessels ≤ 25%; 2 points—expression on 26–50% of cells, 3 points—expression on 51–75% of cells, 4 points—expression on more than 75% of the evaluated structures). Morphometric analysis was carried out on scanned histology slides using the Aperio ImageScope v12.3.3 software.

In the case of the SARS-CoV-2 spike protein expression with a score of >3 (expression score on the endothelium + expression score on the inflammatory infiltrate), an additional immunohistochemistry test was performed with antibodies to ACE2, CD16 and a reverse-transcription droplet digital PCR (RT-ddPCR) assay (RT-ddPCR assay) of the myocardial tissue in accordance with the recommendations of the China CDC Center for the N gene and the Charite Clinic for the E gene (n = 5 including three patients with lymphocytic myocarditis and two patients without myocarditis) [8].

### 2.3. Immunofluorescence Test

Immunofluorescence microscopy (IFM) was performed on myocardium paraffin sections in one patient from the subgroup with myocarditis and one patient from the subgroup without myocarditis (where the expression of the SARS-CoV-2 spike protein was evidenced by the IHC score of >3) with the following antibody cocktail: SARS-CoV-2 spike protein/CD16, SARS -CoV-2 spike protein/CD68, CD80/CD163. Alexa Fluor 594 and Alexa Fluor 488 (Thermo Fisher Scientific, Waltham, MA, USA) were used as secondary antibodies. Sections were counterstained with DAPI (Appli-Chem, Gary, IN, USA). Micrographs were taken with a Leica DM6000B microscope.

### 2.4. Statistical Analysis

The statistical analysis of the acquired data was carried out using NumPy, SciPy, Pandas and Matplotlib libraries in the Python programming language. The Kolmogorov-Smirnov test was used as a normality test. Normally distributed scores were presented as mean values with a confidence interval, while non-normally distributed variables were presented as medians and 25th and 75th percentile values. When comparing scores between groups, the Student’s *t*-test, a permutation test, or a non-parametric Mann–Whitney U-test were used. The differences were considered statistically significant if *p* < 0.05. The Fisher’s exact test was used to compare the frequency of data in groups.

## 3. Results

### 3.1. Subgroup of Subjects with Chronic Lymphocytic Myocarditis

In most patients, the acute phase of coronavirus infection was mild or moderate. In 9 (31%) patients, there were no CT-scan data obtained in the acute period; in the rest of the patients, the median percentage of lung tissue damage was 12.5 (0; 31.5) %. Only in two (7%) patients, the percentage of lung parenchymal lesions on CT scans was >50% (56% and 60%).

In the subgroup of patients with chronic lymphocytic myocarditis, deterioration in the course of pre-existing HF after COVID-19 was registered in 9 (31%) patients, and the onset of HF after COVID-19 was observed in 13 (45%) patients. In 6 (21%) patients with HF diagnosed before COVID-19, there was no deterioration in the clinical manifestations of HF. Only one patient was diagnosed with HF neither before nor after the coronavirus infection.

The most common type of rhythm and conduction disturbances in this subgroup was atrial fibrillation, which was revealed in 7 (24%) patients. Left bundle branch block, ventricular tachycardia, and premature ventricular contractions were detected in 5 (17%), 4 (14%), and 4 (14%) patients, respectively. Other rhythm and conduction disturbances were diagnosed only in a few cases.

Four (14%) patients preserved LVEF, while in 25 (86%) patients, the LVEF was reduced with a median value of 26.5% (25% and 75% quartiles: 23%; 30%). In this group, EMBs were performed from 3 weeks to 17 months after the most recent positive PCR test for SARS-CoV-2 (nasopharyngeal swab) with a median value of 4 (25% and 75% quartiles: 2; 8) months.

Five (17%) patients from this subgroup had a history of vaccination against SARS-CoV-2: Three patients were diagnosed with borderline lymphocytic myocarditis (CoviVac: n = 2; Sputnik V: n = 1), and two with active lymphocytic myocarditis (Sputnik V: n = 2). Nevertheless, there was no evidence of any clinical association between the immunization and the development of myocarditis in these patients. Although the time interval from the vaccination to the development of cardiac symptoms that required the EMB ranged from 1 to 4.5 months (mean value = 2.7 months), which did not exclude the possibility of developing post-vaccination myocarditis, all these patients were found to have VP1-EntV expression in their cardiomyocytes. Therefore, the role of the SARS-CoV-2 vaccine administration in the development of myocarditis in these patients is highly unlikely.

In all studied EMBs, chronic borderline or chronic active lymphocytic myocarditis was diagnosed and confirmed by immunohistochemistry findings. Only five (17%) patients in this group did not demonstrate myocardial expression of VP1-EntV. Among them, only one patient showed weak expression of SARS-CoV-2 spike protein on the inflammatory infiltrate with a score of 1 point. The main parameters of immunohistochemistry are presented in Table 2.

### 3.2. Subgroup of Subjects without Chronic Lymphocytic Myocarditis

The acute phase of coronavirus infection in most patients was mild or moderate. In 2 (22%) patients, there were no CT-scan data obtained in the acute period; in the rest of the patients, the median percentage of lung tissue damage was 25 (10; 29) %. Only in one (11%) patient was the percentage of lung parenchymal lesions on CT > 50% (60%).

In the subgroup of patients without myocarditis, deterioration in the course of pre-existing HF after COVID-19 was registered in only one (11%) patient, and the onset of HF after COVID-19 was observed in 7 (77%) patients. In only one patient did HF not become more severe after the coronavirus infection.

The most common type of rhythm and conduction disturbance was ventricular tachycardia, which was observed in 3 (33%) patients. Atrial fibrillation was detected in only one (11%) patient.

The left ventricular ejection fraction (LVEF) was preserved in only one patient (11%), while in the remaining eight cases (89%), the LVEF was reduced, with a median value of 25 (23; 30) %. In this group, EMBs were performed 3 weeks—20 months after the most recent positive SARS-CoV-2 PCR test (nasopharyngeal swab) with a median value of 5 (2; 9) months.

Five (55%) patients from this group were vaccinated against SARS-CoV-2 using CoviVac or Sputnik V vaccines in 1 and 4 cases, respectively. The time from vaccination to the EMB ranged from 2 to 8 months with a mean value of 4.6 months.

**Figure 1:**
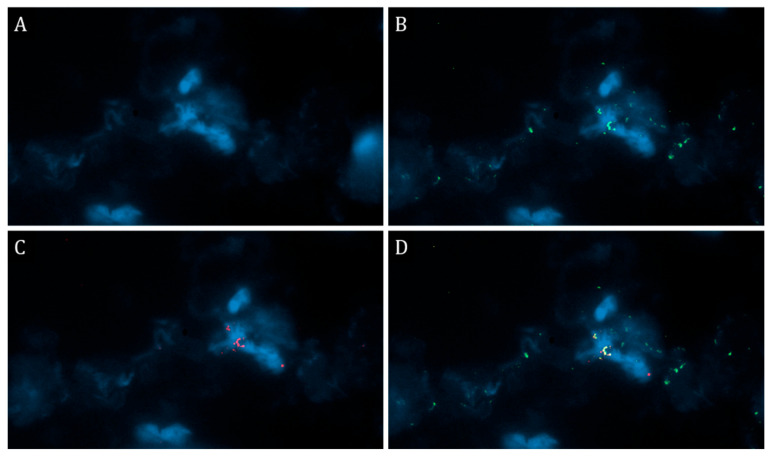
Immunofluorescent examination of the myocardium with a cocktail of antibodies against the SARS-CoV-2 spike protein/CD16. (**A**)—DAPI, (**B**)—green fluorescence of the SARS-CoV-2 spike protein, (**C**)—red fluorescence of CD16, (**D**)—yellow or orange fluorescence corresponding to co-localization of the SARS-CoV-2 spike protein and CD16 on the monocyte membrane; ×630.

In cases where the expression of SARS-CoV-2 spike protein was detected on the endothelium and on the inflammatory infiltrate with a score of >3 (n = 5), we performed PCR tests on myocardial paraffin blocks. In all cases, PCR tests did not detect SARS-CoV-2 RNA. Additionally, we performed an immunofluorescence test with a cocktail of antibodies to SARS-CoV-2 spike protein/CD68 and SARS-CoV-2 spike protein/CD16 in these cases and revealed co-localization of the SARS-CoV-2 spike protein and CD16 expression on the membrane of individual infiltrate cells. We did not detect any expression of the SARS-CoV-2 spike protein in cardiomyocytes (Figure 1).

**Figure 2:**
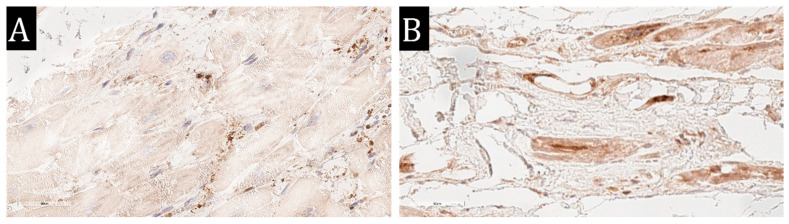
Immunohistochemistry of the EMB specimen obtained from a PASC patient with chronic lymphocytic myocarditis. (**A**)—SARS-CoV-2 spike protein expression in endothelium and perivascular cells; (**B**)—ACE 2 expression in endothelium and perivascular cells, ×400.

We also conducted immunohistochemistry for ACE 2 and found its expression on few vessels obtained during the EMB. Interestingly, we also revealed the SARS-CoV-2 spike protein expression on the endothelium of some intramyocardial vessels in these cases (see Figure 2).

**Figure 3:**
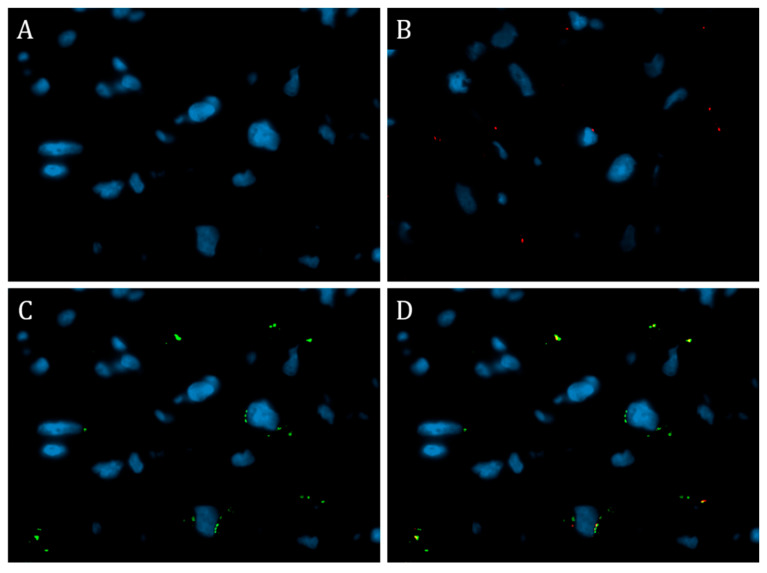
Immunofluorescent test of the EMB specimen from a patient with myocarditis using a cocktail of antibodies against CD163/CD80. (**A**)—DAPI, (**B**)—green fluorescence of CD163, (**C**)—red fluorescence of CD80, (**D**)—green and red fluorescence on the membrane of M2 and M1 macrophages; ×630.

In one case from the subgroup of patients with myocarditis and one case from the subgroup of patients without myocarditis demonstrating the SARS-CoV-2 spike protein expression (the total score of 3 in the endothelium and infiltrate cells), an immunofluorescent test with a cocktail of anti-CD80/CD163 antibodies was performed to determine the M1/M2 ratio of macrophages in the myocardial tissue. The test demonstrated the predominance of M2 macrophages over M1 in the myocardial tissue in both cases (Figure 3).

The statistical data analysis showed that higher levels of the Ang1 expression were found in blood vessels in the absence of myocarditis compared to patients with active lymphocytic myocarditis (*p* = 0.001). However, no statistically significant differences were observed in the expression of this marker between the PASC group and the comparison group. There were also no statistically significant differences in the expression of endothelial activation markers such as VEGF, VWF, and C1q between PASC patients and patients in the comparison group. There were no statistically significant differences between the groups regarding the presence or absence of the SARS-CoV-2 spike protein expression, or changes in the expression of other markers. The percentage of lung tissue damage (severity of the acute coronavirus infection) did not correlate with the increase in the HF severity during the post-acute phase of the coronavirus infection.

There was a statistically significant increase in the HF severity (*p* < 0.05) in patients with atrial fibrillation and ventricular tachycardia compared to patients who had other heart rhythm or conduction disturbances.

**Figure 4:**
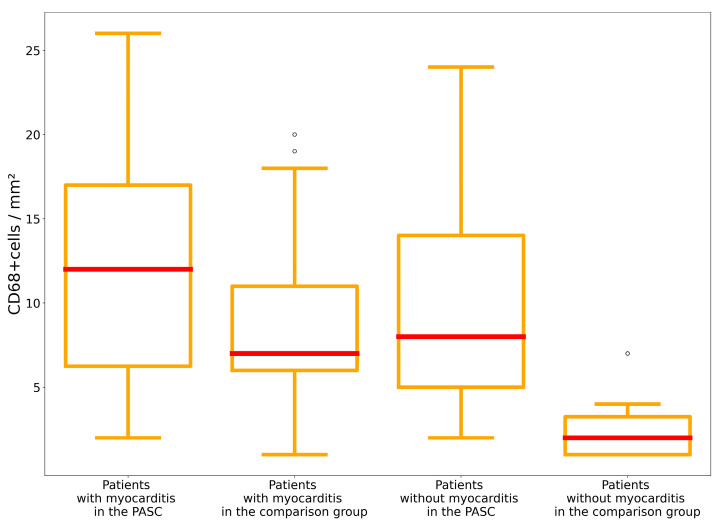
Comparative analysis of the amount of CD68+ macrophages in the PASC and comparison groups.

**Figure 5:**
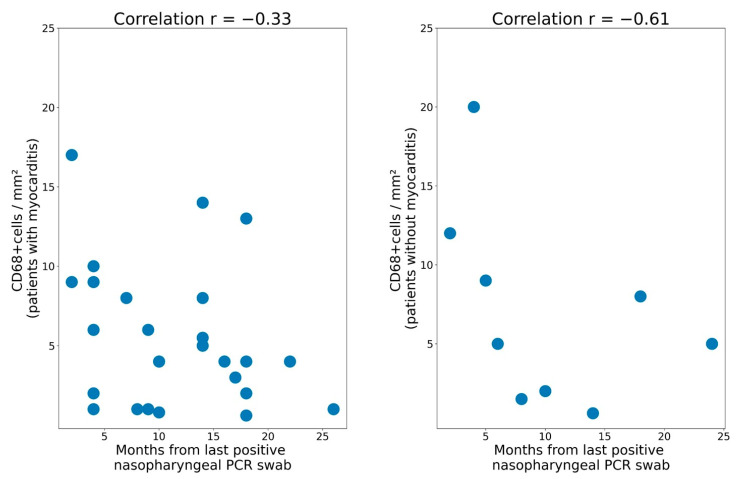
Correlation between the time since the most recent positive PCR test (nasopharyngeal swabs) and the amount of macrophages in the myocardial stroma.

There was an increase in the number of CD68+ macrophages per mm^2^ of myocardium in patients during the post-acute phase of the coronavirus infection compared to patients with no history of COVID-19 (*p* = 0.014 for patients with myocarditis, *p* = 0.007 for patients without myocarditis). An increase in the number of CD68+ macrophages was more often observed in patients with a shorter time interval from the most recent positive SARS-CoV-2 PCR test to the biopsy (r = −0.33 for patients with myocarditis and r = −0.61 for patients without myocarditis) (Figure 4). There was no correlation found between the detection of the SARS-CoV-2 spike protein expression on the endothelium and the time since the last positive PCR test (r = −0.08 for patients with myocarditis and r = −0.29 for patients without myocarditis) (Figure 5).

## 4. Discussion

The development of myocarditis in the presence of SARS-CoV-2 infection and/or after vaccination using RNA fragments of SARS-CoV-2 remains a subject of debate. Data on myocarditis development in patients with COVID-19 are mainly based on MRI and echocardiography findings, as well as detection of a high level of troponin I during the acute phase of the coronavirus infection. Only limited data are available on the EMB findings in patients with suspected myocarditis, although EMB remains the gold standard for in vivo diagnosis of myocarditis [9]. In most described cases, the etiologic role of other viruses could not be ruled out, while the direct effects of the coronavirus infection were not proven. The association of myocarditis development with the acute phase of the coronavirus infection has been the main evidence of SARS-CoV-2 etiology in most studies [10,11,12,13].

However, the obtained data on the COVID-19 pathogenesis remain insufficient. Researchers have suggested several mechanisms that may contribute to the development of myocarditis and other forms of myocardial damage in patients infected with SARS-CoV-2. Among them, direct invasion of the virus into cardiomyocytes, inflammatory reactions or immune response, and microangiopathy have been discussed [14,15,16,17].

A study conducted by Fox SE et al. found that the SARS-CoV-2 virus causes a unique inflammatory response in infected patients, leading to damage to the endothelium. In the heart, such damage can disrupt blood clotting at the level of arterioles, venules, and capillaries, causing thrombosis and, as a result, ischemia and reperfusion. Additionally, endothelial damage may attract non-classical monocytes, activating the complement pathway and causing macrophage-initiated apoptotic damage [18].

Some studies have shown a significant increase in CD68+ macrophages in myocardial tissue during the acute period of the coronavirus infection [18,19].

In our study, we also observed a persistent increase in the number of CD68+ macrophages in PASC; however, the macrophage population was heterogeneous. These macrophages are conditionally divided into different subtypes, including M1 and M2 macrophages. M1 macrophages are considered pro-inflammatory, while M2 macrophages are believed to be anti-inflammatory. Macrophage differentiation is determined by external stimuli, e.g., bacterial lipopolysaccharides can induce macrophage differentiation to the M1 phenotype, while IL-4 can induce macrophage differentiation to form M2 cells [20].

It is known that in addition to cardiomyocytes, resident macrophages are present in the heart and play an important role in its functioning. Studies show that as a result of myocardial ischemia, the number of macrophages increases, indicating their involvement in the repair of the cardiac tissue [21]. Resident macrophages are formed from embryonic precursors and have the ability to self-renew without the involvement of monocytes [22]. In addition, they differ from monocytes in their functions and expression of certain genes. Resident macrophages of the heart usually have M2 characteristics and express CD206, CD163, CD11b. Their main function under disease conditions is the restoration of myocardium, which occurs through the activation of angiogenesis, migration, and proliferation of fibroblasts [23]. This type of macrophage also expresses a number of anti-inflammatory genes that reduce the inflammatory response in damaged tissue [24]. In addition, M2 macrophages indirectly activate myofibroblasts to produce collagen by secreting TGF-β and fibroblast growth factors, and work together with them to release matrix metalloproteinases (MMPs). Some studies have shown that macrophages can directly contribute to fibrosis by synthesizing type VIII collagen [25]. M1 macrophages can significantly activate the synthesis of metalloproteinases MMP 1, 3, 7, 10, 14, and 25, and secrete a range of chemokines and cytokines such as IL-1β, IL-6, tumor necrosis factor-alpha (TNF-α), and chemokine (CC-motif) ligand (CCL)-3 and CCL-4 to enhance inflammation and activate cardiomyocytes and endothelial cells [26]. Moreover, it has been shown that during infection with COVID-19, increased secretion of most of these cytokines was also determined [27]. In intact myocardium, we should see a predominance of M2 macrophages over M1 macrophages. In our study, we documented an increase in the number of CD68+ macrophages mainly due to CD163+ M2 macrophages. In two cases of immunofluorescence microscopy, we also clearly observed a shift in the M1/M2 macrophage ratio in favor of anti-inflammatory M2 macrophages. These changes are associated with the repair of the consequences of cytokine imbalance and angiopathy in the acute phase of coronavirus infection, which leads to post-acute sequelae of SARS-CoV-2 infection (PASC), as evidenced by the decrease in the expression level of endothelial activation markers such as VEGF, VWF, Ang1, and C1q. In our previous study, we showed that the expression levels of these markers increase in the acute phase of coronavirus infection [28]. This is consistent with the literature data that myocardial damage in the acute phase of the coronavirus infection is due to systemic manifestations of cytokine storm and generalized angiopathy [19,29,30,31]. Moreover, a hypothesis that PASC is only associated with the recovery from the angiopathy consequences is supported by the fact that in some patients, cardiac manifestations of PASC disappear spontaneously. It is also important to note that our study demonstrated a positive correlation between the time since the patient’s most recent positive PCR test (nasopharyngeal swabs) and the number of CD68+ macrophages in the myocardial tissue. This may indirectly indicate that in some cases, there is restoration of the myocardial tissue with complete morphological recovery following the acute phase of the coronavirus infection [32].

We believe that the main clinical symptoms of PASC (cardiovascular symptoms) are also due to a local cytokine imbalance and the consequences of angiopathy. Thus, pain can be explained by the effect of cytokines on myocardial nociceptors, arrhythmias related to a local cytokine imbalance with excessive local activation of cardiomyocytes and the formation of reentry foci, dyspnea, and reduced exercise tolerance, as well as transient cardiac dysfunction due to the consequences of angiopathy and ischemic damage to the myocardium.

The direct etiologic role of the coronavirus in the development of myocarditis was not proven in any of the cases in our study. RT-PCR tests did not detect SARS-CoV-2 RNA in any of the analyzed cases. Immunohistochemical expression of SARS-CoV-2 spike protein was only detected in the inflammatory infiltrate in the myocardial tissue and on the endothelium of individual vessels.

It is widely known that the main pathways for SARS-CoV-2 entry into the cell are the ACE 2 and CD16 receptors. Immunofluorescence analysis with a CD16/SARS-CoV-2 spike protein cocktail confirmed that viral antigens and CD16 are located on the cell membrane. The ACE 2 receptor is sparsely distributed in the myocardial tissue and is predominantly detected on pericytes [33].

We observed expression of both ACE 2 and SARS-CoV-2 spike protein on the same myocardial vessels, which confirms the possibility of virus infection of pericytes with subsequent pericyte-associated angiopathy, which, according to our data, was expressed by clear endothelial activation in the acute phase of the coronavirus infection [34]. In some cases of PASC, residual activation of the endothelium was observed with expression of MHC1, VWF, Ang1, VEGF, C1q.

Preservation of SARS-CoV-2 spike protein expression and the absence of virus RNA based on PCR testing suggest that we are only observing viral epitopes on cell membranes while the virus itself is eliminated from tissues during the post-acute phase of the coronavirus infection. However, we cannot definitively state whether there are minimal amounts of viral particles remaining in myocardial tissue that cannot be detected through quantitative PCR.

In most patients included in our study, there was a clear association between the coronavirus infection and the manifestation of symptoms of myocarditis. However, only one patient was diagnosed with active lymphocytic myocarditis and had no expression of VP1-EntV but showed weak expression of the SARS-CoV-2 spike protein in the inflammatory infiltrate with a score of 1. The low level of the SARS-CoV-2 spike protein expression in this case and the presence of VP1-EntV expression in most other patients makes it highly unlikely for COVID-19 to induce the development of coronavirus-associated myocarditis. Moreover, it indicates that the majority of lymphocytic myocarditis cases were chronic and only clinically presented in the post-acute phase of the coronavirus infection. Therefore, SARS-CoV-2 can reasonably be considered a trigger for the development of myocarditis.

The etiologic role of coronavirus in the development of virus-negative inflammatory cardiomyopathy remains unclear. The role of pyroptosis in the development of autoinflammation and its connection to the pathogenesis of virus-negative myocarditis is actively being studied [35].

**Figure 6:**
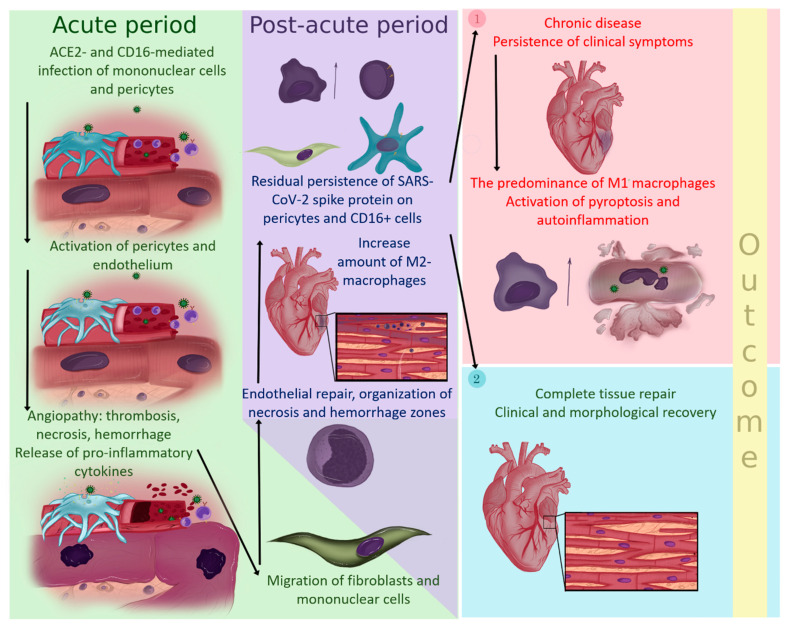
Suggested pathogenesis of SARS-CoV-2-related effects on the myocardium.

M1 macrophages in the myocardial tissue may contribute to the synthesis of NLRP3 inflammasomes, triggering programmed necrosis of cardiomyocytes. In turn, the abundance of DAMPs released into the surrounding tissue may promote the migration and differentiation of monocytes into M1 macrophages, which can lead to the formation of a vicious cycle of disease. These mechanisms require further studies and deeper understanding of the pathophysiology of these processes. Our view on the pathogenesis of the post-acute coronavirus syndrome is illustrated in Figure 6.

## 5. Conclusions

PASC manifestations in the myocardium are characterized by a significant increase in the number of CD68+ macrophages and a decrease in the expression score of markers associated with angiopathy. No evidence of coronavirus-associated myocarditis was observed in any PASC patient. At the same time, coronavirus infection antigens in myocardial tissue may persist indefinitely. Cardiac symptoms of PASC may be explained by a local cytokine imbalance in the myocardial tissue and transient cardiac dysfunction due to acute infection-associated angiopathy.

## 6. Study Limitation

In this study, we could not take into account the possible subclinical course of acute coronavirus infection in our patients, and the possibility of a PCR-negative course of coronavirus infection in patients undergoing diagnostic evaluation and EMB procedures.

## Figures and Tables

**Table 1:** Parameters of patients with the PASC and in the comparison group.

Patient’s Parameters	Patients with Myocarditis with the PASC (n = 29)	Patients with Myocarditis in the Comparison Group (n = 33)	Patients without Myocarditis with the PASC (n = 9)	Patients without Myocarditis in the Comparison Group (n = 8)
Age, year (50%, 25%, and 75% percentiles)	35 (32; 44)	38 (30; 45)	42 (38; 46)	41 (36; 48)
Clinical suspicion of myocarditis (n, %)	29 (100%)	33 (100%)	9 (100%)	41 (100%)
Hs-cTnT > 100 ng/L (n, %)	5 (17%)	7 (21%)	0	1 (13%)
Clinic of chronic heart failure (n, %)	23 (79%)	27 (82%)	8 (89%)	8 (100%)
Ventricular rhythm disorders (n, %)	6 (21%)	9 (27%)	3 (33%)	2 (25%)
Association with viral infection (n, %)	29 (100%)	30 (91%)	9 (100%)	6 (75%)
Association with vaccination (n)	0	0	0	0
Left ventricular ejection fraction at initial examination (n, 25% and 75% percentiles)	28 (23; 33) %	26 (20; 34) %	27 (23; 30) %	26 (22; 38) %
Presence of a clinical phenotype of dilated cardiomyopathy (n, %)	12 (41%)	17 (52%)	7 (78%)	4 (50%)

**Table 2:** Median values of immunohistochemical parameters in the EMB specimens of PASC patients.

Immunohistochemical Markers	Patients without Myocarditis (n = 9)	Patients with Myocarditis Total (n = 29)	Patients with Borderline Myocarditis (n = 10)	Patients with Active Myocarditis (n = 19)
CD3 + cells/mm^2^	4.0 (3.0; 5.0)	19.0 (13.0; 27.0)	11.5 (8.0; 12.25)	20.0 (16.5; 31.0)
CD68 + cells/mm^2^	8.0 (5.0; 14.0)	11,5 (8.75; 14.5)	11.0 (8.25; 15.5)	12.0 (10.0; 17.5)
HLA-DR (score)	1.0 (0.0; 3.0)	3.0 (2.75; 4.0)	3.0 (2.25; 4.0)	3.0 (3.0; 4.0)
MHCI (score)	4.0 (4.0; 4.0)	4.0 (4.0; 4.0)	4.0 (4.0; 4.0)	4.0 (4.0; 4.0)
C1q (score)	1.0 (1.0; 2.0)	3.0 (2.0; 4.0)	2.0 (2.0; 3.0)	3.0 (2.0; 4.0)
VP1-EntV in vessels, score	0.0 (0.0; 0.0)	1.0 (1.0; 2.0)	1.0 (1.0; 2.0)	1.0 (0.0; 1.5)
VP1-EntV in cardiomyocytes, score	0.0 (0.0; 0.0)	2.0 (1.0; 2.0)	1.5 (1.0; 2.0)	2.0 (1.0; 2.0)
VWF in vessels, score	1.0 (1.0; 1.75)	1.0 (1.0; 2.0)	1.0 (1.0; 2.0)	1.0 (1.0; 1.5)
VWF in cardiomyocytes, score	0.0 (0.0; 0.0)	0.0 (0.0; 0.0)	0.0 (0.0; 0.0)	0.0 (0.0; 0.0)
Ang1 in vessels, score	4.0 (4.0; 4.0)	3.0 (2.0; 4.0)	4.0 (3.0; 4.0)	2.0 (1.0; 2.5)
Ang1 in cardiomyocytes, score	0.0 (0.0; 0.75)	3.0 (2.0; 4.0)	2.0 (2.0; 3.0)	3.0 (2.0; 4.0)
VEGF in vessels, score	2.5 (1.25; 3.75)	3.0 (3.0; 4.0)	3.0 (3.0; 3.0)	4.0 (3.0; 4.0)
VEGF in cardiomyocytes, score	0.0 (0.0; 0.0)	0.0 (0.0; 0.0)	0.0 (0.0; 0.0)	0.0 (0.0; 0.0)
SARS-CoV-2 spike protein in infiltrate cells, score	2.0 (1.0; 2.0)	1.0 (0.0; 1.25)	0.0 (0.0; 1.0)	1.0 (0.0; 1.25)
SARS-CoV-2 spike protein in endothelium, score	1.0 (0.0; 1.0)	0.0 (0.0; 1.0)	0.0 (0.0; 1.0)	0.0 (0.0; 1.0)

The values in parentheses are 25% and 75% percentiles. The table provides information on two main subgroups of the study: patients with and without myocarditis (columns 2 and 3). It also includes more detailed information on the third column, which represents patients with active and borderline myocarditis within the subgroup of patients with myocarditis (columns 4 and 5).

## Data Availability

Availability of data and materials. If you need clarifications, or need additional information about the study data, you can write to the email: doctormakarovia@gmail.com.

## Abbreviations

ACE 2	angiotensin-converting enzyme 2
AF	atrial fibrillation
EMB	endomyocardial biopsy
HF	heart failure
IFM	immunofluorescence microscopy
IHC	immunohistochemistry
LVEF	left ventricular ejection fraction
PASC	post-acute sequelae of SARS-CoV-2 infection
VWF	von Willebrand factor
